# Moving Model Test of High-Speed Train Aerodynamic Drag Based on Stagnation Pressure Measurements

**DOI:** 10.1371/journal.pone.0169471

**Published:** 2017-01-17

**Authors:** Mingzhi Yang, Juntao Du, Zhiwei Li, Sha Huang, Dan Zhou

**Affiliations:** 1 Key Laboratory of Rail Traffic Safety (Central South University), Ministry of Education, Changsha, Hunan Province, China; 2 CRRC QINGDAO SIFANG CO., LTD, Qingdao, Shandong Province, China; Universitat Zurich, SWITZERLAND

## Abstract

A moving model test method based on stagnation pressure measurements is proposed to measure the train aerodynamic drag coefficient. Because the front tip of a high-speed train has a high pressure area and because a stagnation point occurs in the center of this region, the pressure of the stagnation point is equal to the dynamic pressure of the sensor tube based on the obtained train velocity. The first derivation of the train velocity is taken to calculate the acceleration of the train model ejected by the moving model system without additional power. According to Newton’s second law, the aerodynamic drag coefficient can be resolved through many tests at different train speeds selected within a relatively narrow range. Comparisons are conducted with wind tunnel tests and numerical simulations, and good agreement is obtained, with differences of less than 6.1%. Therefore, the moving model test method proposed in this paper is feasible and reliable.

## Introduction

Due to the advantages of high speed and efficiency, high-speed railways have become an important mode of transportation, drawing increasing attention worldwide [1]. According to references, worldwide high-speed railway lines will reach 0.59 million kilometers by the end of 2025. Although China started relatively recently, it has the largest rail network in the world after 10 years of development and is expected to reach a total mileage of over 0.3 million kilometers by the end of 2020 [2,3].

However, as train speed increases, the aerodynamic drag increases dramatically; this drag accounts for 85% of the total resistance when the train speed reaches 300 km/h [4,5]. Substantial resistance not only leads to high energy consumption but also demands higher requirements of the train traction system, which negatively impacts further increases in speed [6,7]. Statistics show that 16% of the total energy consumed in the United States is used to overcome aerodynamic drag in transportation systems [8]. Baker notes that an x% reduction of a train’s aerodynamic drag can reduce fuel consumption by 0.5x% [9]. Therefore, the aerodynamic drag of the train is the main method for reducing the overall resistance and energy consumption of high-speed trains [10].

The train aerodynamic drag consists of pressure drag and viscous drag. Because the length-to-width ratio of a train ranges from 50~100, which is much larger than that of any other ground vehicle, the aerodynamic characteristics are more complex than those of cars, trucks or airplanes [11–13]. Full-scale field tests, model tests and numerical simulations are the main methods used to study the aerodynamic properties of a train. The full-scale field test method uses the coasting test principle to measure the train speed and to calculate the train resistance indirectly; however, this method is costly and easily affected by environmental factors, making it unsuitable for the early development and shape design phase of high-speed train manufacturing [14]. The wind tunnel test is the most popular and important method and offers relatively high reliability and repeatability; both domestic and international scholars have performed experiments using this method and have obtained many achievements [15–17]. However, because the train model is stationary and fixed on the floor, this model cannot simulate the relative movement of the train model, the air and the surroundings. The main problem with wind tunnel testing is how to properly simulate the ground [18–19]. Baker and Brockie tested wind tunnels with several different ground simulation methods and found that the measured resistance was usually more than 10% [20]. Air suction floor technology, conveyer belt floor technology [21,22], the double-model method with symmetric mirror deployment [23], or the screen method [24,25] are typically used to alleviate the influence of the negative boundary layer effect on the train aerodynamic drag measurement. However, the ground effect cannot be eliminated completely and additional facility effects may interfere with the tunnel flow itself, decreasing the measurement accuracy.

The developed moving model test rig in which the train model runs along a track can more accurately simulate the relative movement between the train, the ground and the surroundings and has no interference from any floor boundary layer [26–28]. Currently, moving model rigs used for high-speed train aerodynamic studies are mainly located at the Derby railway research center in the UK [29,30] and at Central South University in China. Studies using these rigs have measured the train aerodynamic characteristics including transient pressure, micro-pressure wave and aerodynamic sound pressure level; however, none of these studies measured the aerodynamic drag due to the rapid movement of the train model. This paper proposes a new moving model test method to measure the train aerodynamic drag, which will provide a new and valuable resource for studies of high-speed train aerodynamic properties.

## Test Method Principle

The test principle of the train resistance coefficient based on the moving model is similar to the coasting experiment principle of a full-scale field test. The total resistance of the train can be expressed using different equations, among which the Davis equation is widely accepted and applied [23,31,32]. Generally, this equation is expressed as follows:

$$R=A+BV_{t}+CV_{t}{}^{2}$$ (1)

where *R* is the total operation resistance of the train, *V*_*t*_ is the train speed, and *A* (N), *B* (N·s/m), and *C* (N·s^2^/m^2^) are the coefficients generally obtained from the coasting experiment. The first term, *A*, is the mechanical operation resistance caused by the wheel and track friction and is proportional to the train mass. *BV*_*t*_ is all other mechanical resistance, including transmission loss, brake resistance, and air momentum resistance. When a train operates normally, the circulating engine heat, the cooling system, and the onboard air-conditioner all take in and discharge air, causing air momentum resistance that is proportional to the train velocity. *CV*_*t*_^*2*^ is the train aerodynamic drag and is proportional to the square of the train speed.

When the train model is ejected at a certain speed, and no additional power or air discharge is provided, the movement of the train follows Newton’s second law, which can be presented as:

$$Ma=A+BV_{t}+CV_{t}^{2}$$ (2)

The measurement of the train aerodynamic drag is affected by the Reynolds number. If the Reynolds number reaches 3.6×10^5^, which means that the Reynolds number enters the self-simulation range, then the influence of the Reynolds number on the train aerodynamic characteristics is very small and can be neglected [7]. Because the train model scale used in the test is 1:16.9, the train speed should be over 96 km/h to reach the self-simulation range. Therefore, two measures were taken to minimize the influence of the Reynolds numbers:

1. The experimental train speeds are much larger than the critical self-simulation speed.
2. The experimental train speeds are selected within a very narrow range, in which the influence of the Reynolds number can be neglected.

Under these circumstances, by measuring the train model acceleration at different speeds, coefficients *A*, *B*, and *C* can be calculated using Eq (3):

$$\left\{ \begin{array}{l} Ma_{1}=A+BV_{t1}+CV_{t1}^{2}\\ Ma_{2}=A+BV_{t2}+CV_{t2}^{2}\\ Ma_{3}=A+BV_{t3}+CV_{t3}^{2} \end{array}\right.$$ (3)

The value of $CV_{t}{}^{2}$ is equal to the value given in two previous studies [7]:

$$CV_{t}{}^{2}=\frac{1}{2}\rho S_{x}C_{x}V_{t}{}^{2}$$ (4)

The relationship between the equation coefficient *C* and the aerodynamic drag coefficient *C*_*x*_ is:

$$C_{x}=2C/\left(\rho S_{x}\right)$$ (5)

where *S*_*x*_ is the reference area and generally indicates the train cross section and *C*_*x*_ represents the required train aerodynamic drag coefficient.

The mass of the high-speed train model can be measured by a balance. The simplest and most direct way to measure the train model acceleration is to use an acceleration sensor. However, the irreconcilable contradiction is that the acceleration of the train model can reach 50g when it starts to accelerate and brake while the actual acceleration caused by air resistance is much less than 1g. Thus, the acceleration sensor range is much larger than the measured value, as well as the inertia of the train model inertia itself, resulting in huge experimental errors. Therefore, the indirect measurement method should be adopted, that is, calculating the first derivative of velocity versus time.

Then, the acceleration can be obtained from:

$$a(t)=\frac{\text{d}v}{\text{d}t}$$ (6)

Based on the above principle, the train aerodynamic drag coefficient can be obtained by measuring the train speed.

## The Test Method

### The moving model rig

**Fig 1:**
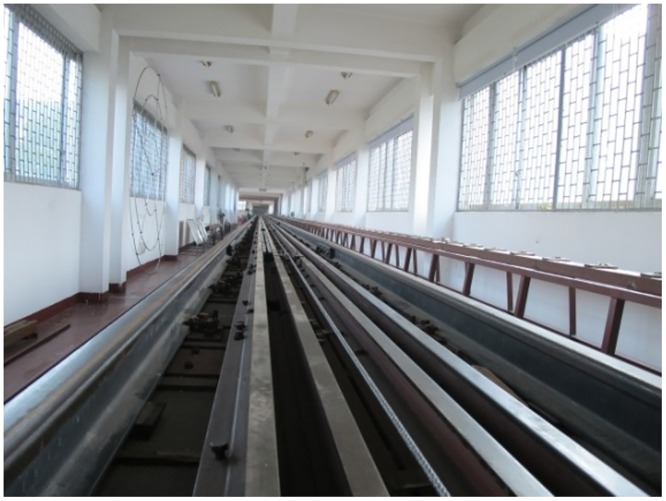
The moving model rig.

The experiments were conducted on the moving model rig at Central South University in China. As shown in Fig 1, the experimental line is a compound line with a total length of 164 m that is divided into an accelerating section, a testing section and a braking section. This moving model system includes the experimental table, power system, acceleration system, control system, test system, brake system, data processing system, and experimental model. The acceleration system of the moving model device uses a multi-level aerodynamic pulley acceleration mechanism, which can achieve a maximum experimental speed of the moving train model of 500 km/h [7]. To prevent electromagnetic disturbance of the onboard system and high impact force, the moving model experimental device uses a mechanical hybrid brake to ensure that there is no damage to the train model or test devices during deceleration and stopping.

### The train model

**Fig 2:**
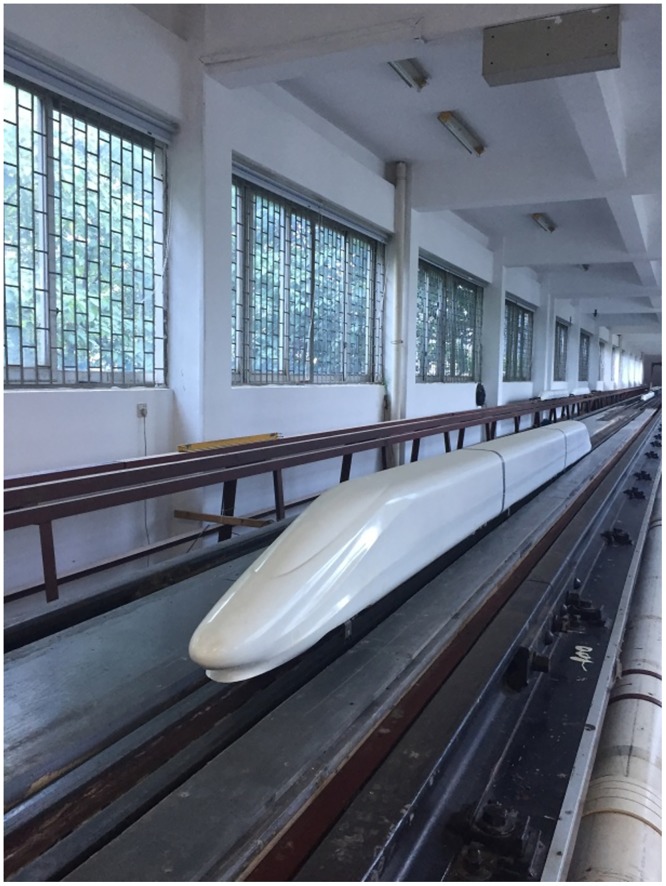
The train model.

**Fig 3:**
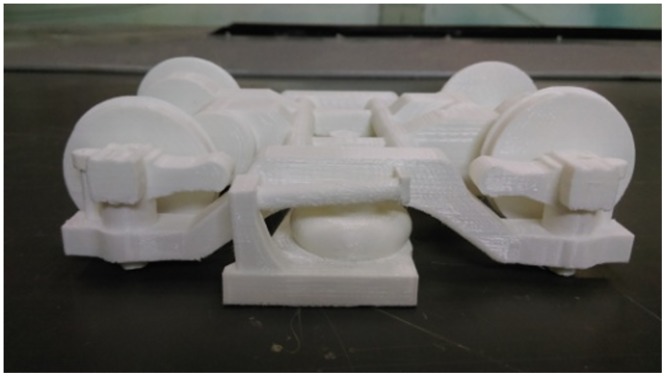
The 3D printed bogie.

As shown in Fig 2, a 1:16.8 scale CRH380A high-speed train model with three coaches was used in the moving model test. The total mass of the train model was 20.16 kg with a length of 4.64 m, a height of 0.198 m and a width of 0.220 m. The model was made of high-strength foam using one-time modeling technology. The model has no pantographs on the top; however, windshields were placed between the inter-carriage gaps, and 3D printed bogies were installed at the bottom of the train model, as shown in Fig 3.

### The speed test system

The train aerodynamic drag is obtained indirectly by measuring the train speed and acceleration. Therefore, the velocity of the train model must be measured properly.

Generally, the velocity of the train model can be measured by an external pilot tube or by photoelectric sensors on the track side or at the bottom of the train model.

For the first method, external pilot tubes are set on top or in front of the train model to measure the train speed directly. This method can collect complete information of the dynamic velocity, but the pilot tubes themselves and their mounting brackets disturb the flow, resulting in additional measurement errors.

The second method is to set up one row of photoelectric sensors on one side of the track and to measure the train arrival times at each of the two sensors. Based on these measurements, the average velocity in the running region are calculated. This method has no contact with the train model and the moving model rig; thus, no interference occurs. However, it requires large number of sensors to acquire sufficient velocity data; too many sensors will result in shorter distances between the sensors, enlarging the errors caused by differences in sensor installation.

The third method uses the photoelectric sensor installed at the bottom of the train model to scan the zebra stripes laid on the track between two rails. The black and white stripes continuously alternate; the white stripes reflect the infrared light back to the sensor, while the black stripes have no reflection. The sensor then converts the optical signal into an electrical signal, generating a series of high and low continuous level pulse signals. Because the interval length between the black and white stripes is constant, the time width of the pulse signal is measured based on the obtained velocity. The method has the advantages of a simple structure and little flow field disturbance; however, it requires high accuracy for the zebra stripe length and a high contrast of the black and white colors.

Based on the characteristics of the three methods above, velocity tests based on the stagnation pressure measurement are proposed in this paper because this method has little flow interference, high accuracy and abundant data.

**Fig 4:**
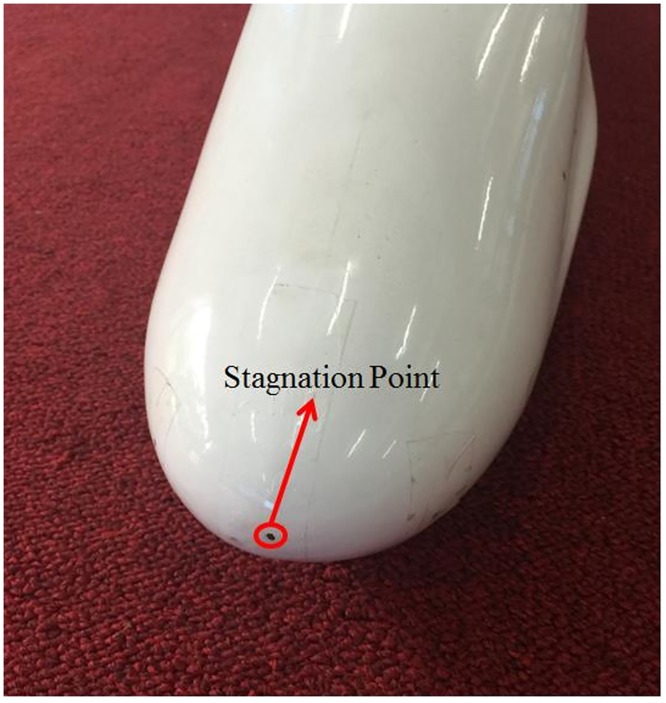
Stagnation point.

The front tip of the high-speed train generally has a high pressure area, and a stagnation point occurs in the center of the region, as shown in Fig 4. The pressure *p* at the stagnation point is equal to the dynamic pressure head of the sensor, which can be expressed as:

$$p=0.5\times \rho \times V_{t}{}^{2}$$ (7)

A pressure sensor is used to directly measure the stagnation pressure *p*; then, the velocity *V*_*t*_ of the train can be calculated by:

$$V_{t}=\sqrt{p/(0.5\times \rho })$$ (8)

Honeywell differential pressure sensors are used in this experiment; their maximum measurement range is 5000 Pa with an accuracy of 0.25% and a response frequency of 10 kHz. The accuracy and frequency are sufficiently large to obtain the pressure information and satisfy the requirements of the experiment.

**Fig 5:**
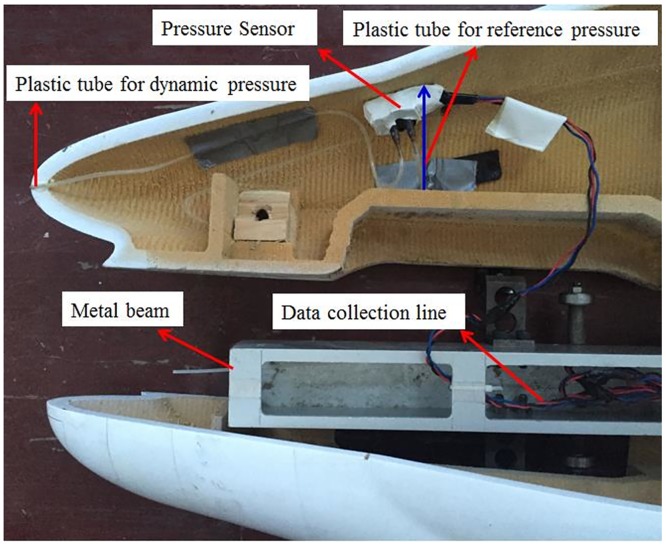
Stagnation pressure test system.

Measuring the stagnation pressure is similar to measuring the surface pressure in a wind tunnel. As shown in Figs 4 and 5, a hole with a diameter of 1.5 mm is opened at the stagnation point of the train head along the normal direction to the model surface. A hollow plastic tube with an outer diameter of 2.0 mm and an inner diameter of 1.0 mm goes through the hole with glue on the surface. One end of the tube maintains the same smoothness as the external surface of the train model, and the other end is connected to the differential pressure sensor. Because too long of a plastic tube will increase the response time, distorting the relationship between pressure and time, the plastic tube should be as short as possible to satisfy the sensor installation requirements for measuring the instantaneous pressure.

The other side of the differential pressure sensor is connected to the reference pressure, which is generally a large, sealed container with a constant pressure. Because the models used in the experiment are too small to use a large container, a sealed, hard plastic tube is used instead. The sealed tube is approximately 5~10 cm long and is fixed vertically on the inner wall of the train model, perpendicular to the moving direction of the train. The direction of the reference pressure tube must not be parallel to the moving direction; in this case, the excessive inertia force generated by the instant acceleration of the train would cause violent fluctuations of the air inside the plastic tube, resulting in large measurement errors.

The differential pressure sensor should follow a similar installation principle and be placed on the inner surface of the train model. If restricted by geometry, the metal beam of the train body could be an alternative location for installing the sensor. However, because the metal beam and the wheel have a hard connection, the train vibrations will pass directly to the metal beam of the train body and to the sensor. Therefore, soft glue should be used to fasten the sensor onto the metal beam to reduce the effect of train vibration.

The sensor converts pressure signals to electric signals in real time, and the electric signals are transmitted to the signal collection board through the data line of the sensor and are then stored in the collection board. The stored signals are transmitted via cables to computers and are finally analyzed. The sensor, data acquisition system and storage system were all installed inside the train model, so that the interference of the whole system to the flow field can be neglected.

## Results and Discussions

### Results of train velocity and acceleration

**Fig 6:**
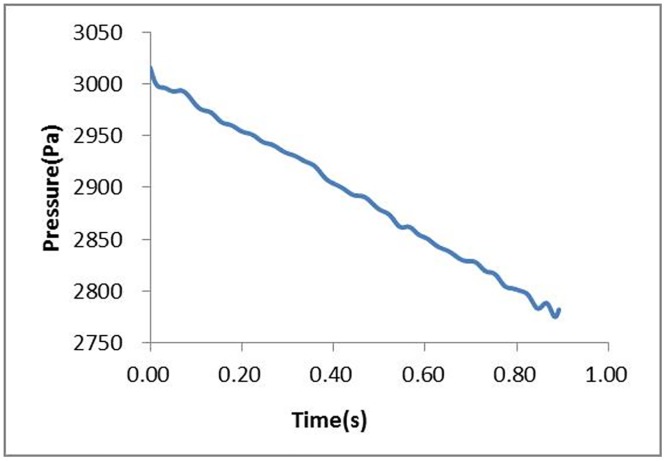
Stagnation pressure of the train.

Fig 6 shows the stagnation pressure curve of the train model moving in the experimental section at a speed of 250 km/h. Due to the existence of train aerodynamic drag and wheel-rail friction drag, the velocity of the train model decreases as the train moves along the track without power. According to Eq (7), the stagnation pressure of the train model decreases with the running time, as shown in Fig 6.

**Fig 7:**
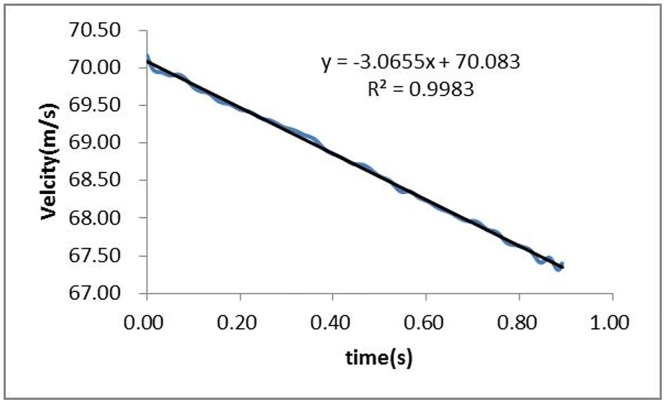
Velocity of the train.

Based on Eq (8), the velocities of the train model are calculated and shown in Fig 7. As shown in the figure, both the velocity and the stagnation pressure decrease linearly over time. Fitting the velocity curve in Fig 7, Eq (9) is obtained to express the relationship between the train velocity and time, and a good correlation of 0.9983 is obtained.

$$V_{t}=-3.0665t+70.083$$ (9)

The first derivative is taken to Eq (9) to obtain the train acceleration, which can be described as:

$$a=dV_{t}/dt=-3.0665$$ (10)

To avoid data variability and to ensure reliability, repeatability tests were conducted. Table 1 lists the initial velocity and the calculated acceleration values of 10 tests at train speeds ranging from 200 km/h to 250 km/h. The average value, standard deviation and relative standard deviation are analyzed and shown in Table 2. The relative standard deviations of the initial speed and the acceleration are 0.68% and 2.27% for the initial speed and acceleration, respectively, which satisfy the engineering requirements. The results show that the test method is reliable and has a high measurement accuracy.

**Table 1:** Repeatability tests.

Measurement Times	1	2	3	4	5	6	7	8	9	10
Velocity (m/s)	70.08	68.96	69.55	69.34	69.70	69.35	69.64	69.58	69.52	70.33
Acceleration (m/s^2^)	3.07	2.90	3.09	3.07	2.96	3.06	3.04	3.01	2.91	3.08

**Table 2:** Velocity and acceleration standard deviations.

	Average Value	Standard Deviation	Relative Standard Deviation
Velocity (m/s)	69.604	0.384	0.55%
Acceleration (m/s^2^)	3.018	0.069	2.27%

**Fig 8:**
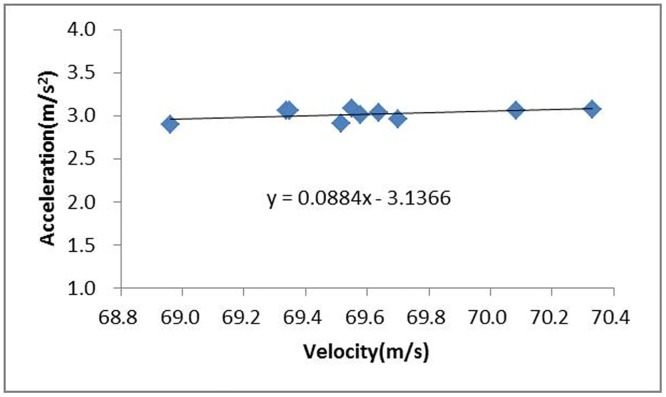
Relationship between the train speed and acceleration.

Because the power of the moving model test system is provided by a rubber rope and because the elastic force of the rope is affected by temperature and motion state, the velocity of the train model may not be exactly match the intended velocity. If the initial speed of the train is 250 km/h, multiple measurements and analyses should be performed to obtain the actual acceleration of the train model at a speed of approximately 250 km/h. Fig 8 gives the relationship between the train speed and acceleration; this curve shows that the acceleration increases gently and slowly with speed. The regression function of the fitted acceleration can be presented as:

$$a=0.0884V_{t}-3.1366$$ (11)

The acceleration of the train model at a speed of 250 km/h can be calculated as 3.002 m/s^2^ using Eq (11). The acceleration of the train model at different train speeds can be calculated similarly.

### Results of the train aerodynamic drag coefficient

The accelerations of the train model at speeds of 240 km/h, 250 km/h and 260 km/h are obtained using the method proposed in this paper, as shown in Table 3.

**Table 3:** Accelerations at different train speeds.

Train Model	Velocity (km/h)	Acceleration (m/s^2^)
CRH380A	240	2.8340
	250	3.0020
	260	3.1675

For the 1:16.8 scale CRH380A train model used in the test, the total mass is 20.16 kg, and the reference area *S*_*x*_ is 0.0394 m^2^. Using these parameters in Eq (3), the resistance of the train can be expressed as:

$$R=19.51027+0.00333V_{t}+0.008456V_{t}{}^{2}$$ (12)

According to Eq (5), the aerodynamic drag coefficient of the train model can be calculated as *C*_*x*_ = 0.3504.

### Results verification

To verify the reliability of the method proposed in this paper, the aerodynamic drag coefficient obtained from the moving model test is compared with results from a wind tunnel test and a numerical simulation.

**Fig 9:**
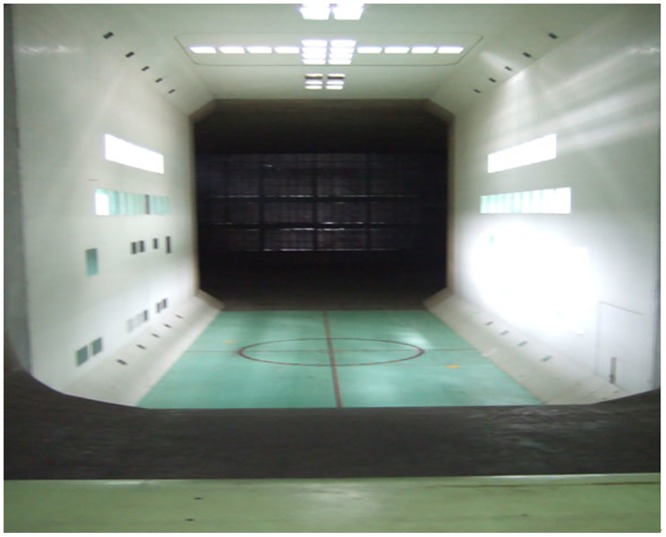
Experimental section of the wind tunnel.

The wind tunnel test was conducted in the wind tunnel in the low-speed aerodynamic institute of the China Aerodynamics Research & Development Centre. As shown in Fig 9, the cross section of the experimental section is 8 m × 6 m, with a length of 15 m, and the wind speed in the tunnel can reach 20~70 m/s. A turntable was placed in the middle of the floor to perform experiments under different wind angles. The front and back edges of the floor are designed with a streamlined shape to minimize flow interference.

**Fig 10:**
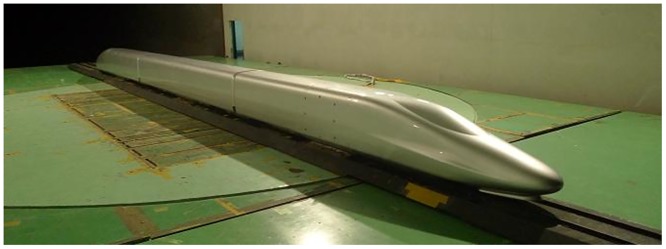
Train model in the tunnel.

**Fig 11:**
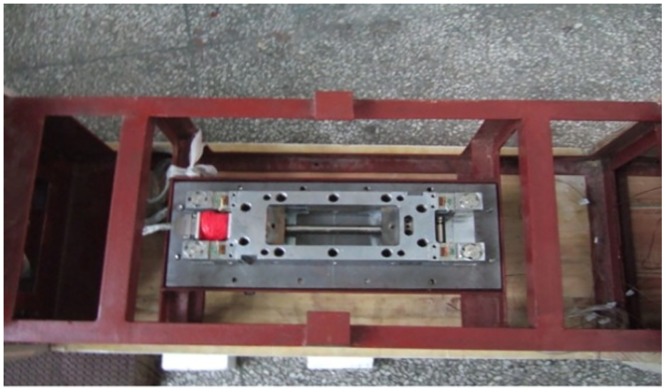
The six-component balance.

A 1:8 scale CRH380A train model consisting of 3 coaches was placed in the middle of the floor, as shown in Fig 10. The aerodynamic drag was measured by a six-component balance that was installed at the bottom of the train model, with one side of the balance fixed to the screen floor by a pole, as shown in Fig 11.

**Fig 12:**
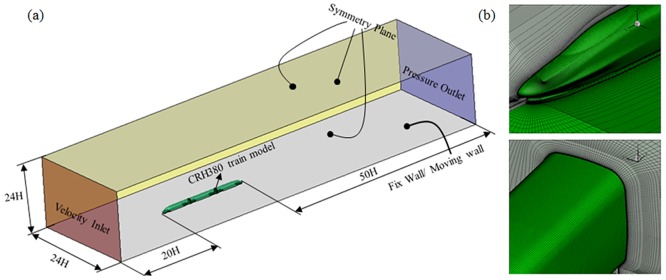
Computational domain and mesh.

In addition, numerical simulations were conducted under different ground wall conditions. As shown in Fig 12(a), the train model is at 1:8 scale and consisted of three coaches. Taking the train height *H* as the characteristic dimension, the computational field velocity inlet is 20*H* away from the train head nose, and the pressure outlet is 50*H* away from the train tail nose. The width and height of the domain are 24*H* and 24*H*, respectively. Fixed wall and moving wall boundary conditions were given to the bottom surface to analyze the influence of the ground effect. Both side surfaces and the upper surface were set as symmetry planes to eliminate the boundary influence.

In this case, a structured grid was built, and the total mesh had 30 million elements. The first layer thickness was 0.5 mm, as shown as Fig 12(b), meaning that the non-dimensional grid thickness fell the range of 30~300. Therefore, the Detached Eddy Simulation model was used to simulate the aerodynamic performance of the CRH380A high-speed train.

Table 4 shows that the results obtained from the moving model test were 6.1% larger than those of the wind tunnel test. This difference is mainly because the train model is stationary on the fixed floor of the wind tunnel, and the fixed floor generates ground effects. These ground effects change the flow field structure at the bottom and rear of the train model, decreasing the train aerodynamic drag. In addition, the wind tunnel test uses a six-component balance, and the support poles for the balance also affect the aerodynamic drag. Finally, the vibration of the model train creates additional errors. The difference in the test results is consistent with the conclusion that different types of wind tunnel ground simulation methods cause no more than 10% of the resistance error [20]. For the numerical simulation method, the results from the moving wall condition are very close to those of the moving model test, while the results from the fixed wall condition are close to those of the wind tunnel test. This finding further verifies the influence of ground effects and shows the advantage of the moving model test method proposed in this paper.

**Table 4:** Results of different experimental methods.

Methods		*C*_*x*_	Difference %
Wind tunnel test		0.330	/
Numerical simulation	Fixed wall	0.316	-4.2
	Moving wall	0.352	6.6
Moving model test		0.350	6.1

## Conclusions

According to the formation mechanism of high-speed train resistance, a new moving model test method based on stagnation pressure measurement is proposed to obtain the aerodynamic drag coefficient of the train. Because a stagnation point occurs in front of the nose of the train model and because the pressure at the stagnation point is equal to the dynamic pressure of the sensor, the velocity can be calculated; then, the first derivation can be taken to obtain the train acceleration. Based on Newton’s second law, the aerodynamic drag coefficient can be resolved using the accelerations at different train speeds. Compared to the traditional wind tunnel method, this method has two apparent advantages. First, this method can simulate the real relative movement between the train, air and ground, avoiding the influence of the ground effect on the aerodynamic drag measurement. Second, the test and acquisition systems are all installed inside the train model, so no flow interference occurs. Comparisons are also conducted with wind tunnel tests and numerical simulations, and good agreement is obtained; therefore, the method proposed in this paper is reliable and effective. Developing and improving this method will provide a new and effective method for train shape optimization and drag reduction.

## References

[1] Takagi R, Dong W. Reviewer of high-speed railway development in recent decades. Western. Technology. 2005;4: 44–58.

[2] Shen Z. High-speed train and key technology. Academic Dynamic Report. 1997;3: 5–14.

[3] Shen Z. The superiorities innovatively developing high-speed train technology in China. Science (China). 2012;57: 594–599.

[4] Gawthorpe R. G.. Aerodynamics of trains in the open air. Railway Engineer International 1978;3: 7–12.

[5] He Q. Some mechanical problems in high speed railways. Science Foundation of China. 1994;3: 30–232.

[6] Lu G. The aerodynamics points of high-speed trains. Railway Vehicles. 2006;44: 1–3, 44.

[7] Tian H. Train aerodynamics. Beijing: China Railway Press; 2007 pp. 160–161.

[8] Wood RM. Aerodynamic drag and drag reduction: energy and energy savings. AIAA Research Paper 2003–0209. Reno, NV: American Institute of Aeronautics and astronautics; 2003.

[9] Baker C. J. Fact and friction. Technology Update. 1983: 86–88.

[10] Peters JL. Aerodynamics of very high speed trains and maglev vehicles: state of the art and future potential. Int J Vehicle Des. 1983;3: 308–413.

[11] Baron A, Mossi M, Sibilla S. The alleviation of the aerodynamic drag and wave effects of high-speed trains in very long tunnels. J Wind Eng Ind Aerodynamics. 2001;89: 365–401.

[12] Schober M, Weise M, Orellano A, Deeg P, Wetzel W. Wind tunnel investigation of an ICE3 end car on three standard ground scenarios. J Wind Eng Ind Aerodynamics. 2010;98: 345–352.

[13] Hemida H, Gil N, Baker C. LES of the slipstream of a rotating train. J Fluids Eng. 2010;132: 1031–1039.

[14] Lukaszewicz P. A simple method to determine train running resistance from full-scale measurements. P I Mech Eng F. J Rai 2007;221: 331–337.

[15] Maeda T, Kinoshita M, Kajiyama H, Yanemoto K. Aerodynamic drag of Shinkansen electric car (series 0, series, 200, series 100). Railway Technical Research Institute, Quarterly Reports 1989;30: 48–56.

[16] Ido A, Iida M, Maeda T. Wind tunnel tests for nose and tail of train. RTRI JNR. 1993;7: 7.

[17] Chen Y, Zhang J. Study of the high speed train aerodynamic drag. China Railway. 1998;2: 43–47.

[18] Schetz JA. Aerodynamics of high speed trains. Annu Rev Fluid Mech. 2001;33: 371–414.

[19] Willemsen E. High Reynolds number wind tunnel experiments on trains. J Wind Eng Ind Aerodynamics. 1997;69–71: 437–447.

[20] Baker CJ, Brockie NJ. Wind tunnel tests to obtain train aerodynamic drag coefficients: Reynolds number and ground simulation effects. J Wind Eng Ind Aerodynamics. 1991;38: 23–28.

[21] Kwon H, Park Y, Lee D, Kim M. Wind tunnel experiments on Korean high-speed trains using various ground simulation techniques. J Wind Eng Ind Aerodynamics. 2001;89: 1179–1195.

[22] Ido A, Kondo Y, Matsumura T, Suzuki M, Maeda T. 2001 Wind tunnel tests to reduce aerodynamic drag of trains by smoothing the under-floor construction. Quarterly Report of RTRI. 2001;42: 94–97.

[23] Brockie NJW, Baker CJ. The aerodynamic drag of high speed trains. J Wind Eng Ind Aerodynamics. 1990;34: 273–290.

[24] Golovanevskiy VA, Chmovzh VV, Girka YV. On the optimal model configuration for aerodynamic modeling of open cargo railway train. J Wind Eng Ind Aerodynamics. 2012;107–108: 131–139.

[25] Kwon H, Nam S, You W. Wind tunnel testing on crosswind aerodynamic forces acting on railway vehicles. J Fluid Sci Technol. 2010;5: 56–63.

[26] Pope CW. The simulation of flows in railway tunnel using 1/25th scale moving model facility. 7th International Symposium on the Aerodynamics and Ventilation of Vehicle Tunnels; 1991. pp. 48–56.

[27] Humphreys ND, Baker CJ. Forces on vehicles in cross winds from moving model tests. J Wind Eng Ind Aerodynamics. 1992;44: 2673–2684.

[28] Sun PP. Research on aerodynamic drag reduction of high-speed train with non-smooth surface. Zhejing: Zhejiang University 2012.

[29] Johnson T, Dalley S. 1/25 scale moving model tests for the trans-aero project In: Schulte-Werning B, Gregoire R, Malfatti A, Matschke G, editors. Transaero-a European initiative on transient aerodynamics for railway system optimization. Berlin: Springer; 2002 pp. 123–135.

[30] Miao X, Li M, Yao Y, Huang Z, Deng Y. Wind tunnel test investigation on the shape optimization of head car of the high speed train. Journal Railway Science and Engineering. 2012;9: 94–98.

[31] Rochard BP, Schmid F. A review of methods to measure and calculate train resistances. P I Mech Eng F. J Rai. 2000;214: 185–199.

[32] Schetz JA. Aerodynamics of high-speed trains. Annu Rev Fluid Mech. 2001;33: 371–414.

